# A study on the mediating-moderate effect of the types of illicit drugs on mental health in China

**DOI:** 10.3389/fpubh.2024.1431854

**Published:** 2024-09-10

**Authors:** Bo Zhou, Jintao Tan, Wenli Li, Cheng Yu

**Affiliations:** ^1^School of Public Administration, Guangzhou University, Guangzhou, China; ^2^Research Institute of Social Development, Southwestern University of Finance and Economics, Chengdu, Sichuan, China; ^3^School of Sociology and Anthropology, Sun Yat-sen University, Guangzhou, Guangdong, China; ^4^Seventh Affiliated Hospital, Sun Yat-sen University, Shenzhen, Guangdong, China

**Keywords:** drug type, mental health, length of drug-use history, drug regulation, mediating moderate effect

## Abstract

**Background:**

In China, over 5 million people have been identified and registered by the public security institutions for using illicit drugs. The aim of this study is to compare the influence of different types of illicit drugs on the self-reported mental health of Chinese people. In particular, we want to assess the damage of Heroin, Methamphetamine and Ketamine to mental health in a social environment where drug use is strictly regulated.

**Methods:**

The study is based on survey with 6,906 people who use drugs in Guangdong province, China. Risk of mental health issue is measured using the Brief Symptoms Inventory 18 (BSI-18) Scale, and a higher BSI-18 score indicates more severe mental health problems. The data was analyzed through multilevel regression analysis, propensity score matching analysis and mediation analysis.

**Results:**

The three major types of illicit drugs have both moderating and mediating effects on the length of drug-use history, that Heroin use leads to longer drug-use duration, while Ketamine use causes more damage on mental health per unit time of drug-use duration. Average duration of Methamphetamine use is 0.7 year shorter than average duration of Heroin use, and average duration of Ketamine use is 1.7 year shorter than average duration of Heroin use. For each year of increase of drug-use duration, Ketamine use leads to 1.2 times more of BSI score increase than Heroin use, and 2.3 times more of BSI score increase than Methamphetamine use.

**Conclusion:**

These three drugs are associated with severe mental health issue in a society with strict drug regulation. Attention should be paid to the mental health of people regardless of the type of drugs they use.

## 1 Introduction

Previous studies have recognized the limitations of laboratory-based analysis of substance addiction and emphasized the influence of social factors (1). Some studies suggest that the risk of substance use is positively correlated with social marginalization. For instance, a cross-national survey conducted by the WHO in Eastern Europe found that drug use is associated with living in a one-parent family, lacking family support, having a low commitment to school, and having poor communication with friends of the opposite sex (2). A study on Turkish college students also shows that residing at home alone and studying abroad would increase the odds of substance use by 1.3 and 2.5 times, respectively (3). On the other hand, some studies have noted the social attributes of addictive substances due to their wide user base, pointing out that social frequency is positively associated with substance use among young people. The decrease in face-to-face peer contact in the early 21st century has led to a decline in adolescent substance use (including cigarette, alcohol, and cannabis) in more than 25 countries (4). Even frequent on-line communication with friends can significantly increase the risk of adolescent substance use (5). In the context of drugs having social attributes, viewing people who use drugs as marginalized individuals and adopting traditional strategies of enhancing social support may not necessarily be effective and may even increase the risk of substance use.

Research on substance use involving social impact generally only considers social factors as influencing factors or treatments, therefore the analysis of social impact is not comprehensive. Yet drug use behavior occurs in a certain social context and may also have different effects on mental health through drug prohibition methods and the image of people who use drugs shaped by propaganda. When analyzing the relationship between drug use and mental health, it is necessary to consider the complex impact of the social background (6). This study chooses to analyze people used drugs in China, which helps to understand the psychological effects that various major drugs will have in a society that is extremely intolerant toward drugs. Most drug intervention programs in Europe and America require voluntary participation and view substance addiction more as a personal issue (7). Compulsory drug rehabilitation interventions has only been adopted for prisoners who use drugs in USA, UK and a few other Western countries (8, 9). Due to the harm caused by opium to Asia in history (10) and the threat posed by the Golden Triangle (the border area of Thailand, Myanmar, and Laos) in recent decades (11), many Asian countries, including China, Singapore and Vietnam, choose to implement compulsory drug rehabilitation and pay more attention to the social damage caused by drug-related crimes (12, 13). When analyzing the psychological effects of drug use in Asian countries, it is necessary to consider that drug use is often defined as an illegal behavior and people who use drugs need to undergo compulsory drug rehabilitation with certain punitive nature (14).

Public security institutions in China have identified and registered 6.79 million people who used illicit drugs in the last decade, among which 1.12 million have engaged in drug use in 2022 (15). A few decades ago, when many Chinese people had insufficient awareness of the harm of drugs, some urban and rural communities even regarded sharing hard drugs (especially Heroin and Methamphetamine) as a form of social interaction (16). Coupled with the promotion of the black industry behind drug dissemination, the spread of drugs in some areas of China was astonishingly fast. During the period when drugs were relatively rampant, convenience stores in some areas sold prohibited drugs, such as cough syrup containing Codeine, and tools for smoking Methamphetamine (i.e., pre-cut tin foil and pipes). Many people in cities started with using cough syrup that could be bought at the school gate during their teenage years, then moved onto using stimulants or Methamphetamine in dance halls (17). In rural areas, many people who use drugs were first exposed to Heroin (often hidden in cigarettes) shared by returned migrant workers from cities (18, 19). In social gatherings, villagers who were not aware of the harm of drugs or had ulterior motives would share Heroin with fellow villagers, spreading drug-use behavior to entire social circles.

There are very few existing studies on the mental health effects of drug use in China, and there are several major issues in the research methods within these studies. First, existing studies mainly rely on small sample interviews or case analyses, often conducting quantitative analysis on a sample less than 300 and highly concentrated geographically (20–22). Second, existing studies in China tend to treat many types of drugs as the same and analyze people who use these drugs as one group. For example, a study conducted in three compulsory isolation institutions in Guiyang city categorized Methamphetamine, Ketamine, and ecstasy as new synthetic drugs and treated them as the same in data analysis (23). Third, existing studies mainly provide references for policy-making by describing phenomena, and there is relatively insufficient analysis of the causes and mechanisms of drug use and its impact (24).

The current study used large sample survey data collected in Guangdong Province, China, to analyze the impact of drug type and length of drug-use history on mental health. In China where drug use is strictly regulated, the duration of drug use is influenced by the addictive nature of the drugs as well as the external forces from public security institutions to cease drug use. Unlike prior studies that ignore the varied effects of different types of drugs (25), this study focuses on the significant differences in the common social settings for the three most prevalent types of drugs in China (15). The consumption of Heroin and Methamphetamine is relatively discreet and can be done in private settings with friends or alone. The consumption of Ketamine, often involves loud music to achieve a pleasurable experience, and is more easily detected and controlled (15). The negative effects of different types of drugs also vary in terms of how each intensifies with prolonged drug-use duration. Therefore, this study goes beyond treating drug-use duration as the only major influential factor on mental wellness (26), and aims to analyze the moderating and mediating roles of drug type in the relationship between length of drug-use history and mental health in a society with strict drug prohibition, in order to better understand the real dangers of drugs outside of laboratory settings and deepen our understanding of psychological outcomes of drug using in a social setting different from the western countries.

## 2 Method

### 2.1 Data

In 2021, our research team conducted a survey on drug-use history and health in Guangdong Province. Guangdong is a coastal province locating in southern China, which possesses the largest amount of people with drug-use history in China (over 0.8 million and around 1/6 of the total number of China). Our survey adopted a stratified mixed sampling method, using a sampling frame from the Narcotics Control Bureau of Guangdong Province consisting of people who used drugs and identified by this institution. First, we randomly select 8 cities in Guangdong based on regional, economic conditions, and demographic characteristics. Then in each city we randomly select 10 sub-districts (*Jiedaos*, communities in urban regions) and 5 townships (communities in rural regions). Finally, we randomly drew 90 cases from each sub-district and 60 cases from each township, aiming for a total of 9,600 respondents. With the assistance of drug rehabilitation social workers in each city, our team conducted face-to-face interviews and obtained a total of 6,906 valid cases. Informed consent to participate in the study has been obtained from all our respondents.

### 2.2 Variables

This study uses BSI-18 score as the dependent variable in the regression models. The Brief Symptom Inventory (BSI-18) consists of 18 questions (27). Each question describes a symptom of a certain psychological issue, and the respondents rate the severity of the symptom from 1 to 5, with a total score ranging from 18 to 90. A higher BSI score indicates more severe mental health problems. Prior studies systematically compared 10 simplified versions of the widely used Symptom Check List 90 (SCL-90) and found that among the many simplified versions, BSI-18 had high validity while reducing the amount of questions by 80%. In order to control the length of the questionnaire, ensure response rate and quality, this study selected BSI-18, which contains only sub-items for somatization, anxiety, and depression aspects from the SCL-90.

The questionnaire of our survey can be divided into four sections, which, respectively, inquire about the demographic basic information of the respondents, drug addiction and rehabilitation situation, drug rehabilitation intervention, and reintegration into society. There are two key independent variables in our regression models, including the type of drug use (1 = Heroin only, *n* = 1,561; 2 = Methamphetamine only, *n* = 2,937; 3 = Ketamine only, *n* = 707; 4 = other, *n* = 1,701) and the duration of drug use. Other independent variables include age, gender, marital status (single, married, divorced), number of child, number of family members, years of education, personal monthly income, community type (0 = urban, 1 = rural) and duration since last drug use.

### 2.3 Statistical analyses

#### 2.3.1 Multilevel regression analysis

We first roughly estimate the impact of independent variables on mental well-being for our whole sample, using a set of multilevel linear models predicting the BSI score. Model 1 only includes demographic variables, duration of drug rehabilitation (compulsory drug rehabilitation program would be implemented on people who are identified to be dependent on drugs by the Narcotics Control Bureau), and types of drugs are considered to be influencing factors. The length of drug-use history is added to the Model 2. Model 3 further includes an interaction term between drug types and duration of drug use.

The regression analysis in this study adopts a 3-level linear model with fixed coefficient random intercepts, with individuals as the first level, community (sub-district/township) as the second level, and cities as the third level. In fact, the selected communities in our study are affected by drugs in markedly different ways due to differences in the level of economic development, urban–rural ratios, location, and the implementation of local anti-narcotics and drug rehabilitation policies. For instance, coastal cities in Guangdong are more severely affected by the Methamphetamine, while it’s more urgent for inland cites to deal with the Heroin from the Golden Triangle, so cities and communities would focus on specific types of drugs in their counter-narcotics advocacy. As a result, Heroin users in inland communities might be confronting greater stigmatization than those in coastal communities, which can lead to different risks of psychological issues for Heroin users with similar drug use patterns. Applying multilevel linear model in this study allows for better control of such potential systematical differences of BSI scores across regions.

The Intraclass Correlation Coefficient of the city level (ICC ranges from 0.017 to 0.025; *p*-value ranges from 0.014 to 0.018) and sub-district/township level (ICC ranges from 0.140 to 0.147; *p*-value ranges from 0.021 to 0.023) are both statistically significant, indicating that using the multilevel models does help reduce the influence of systematic difference across regions.^1^

#### 2.3.2 Propensity score matching

To reduce selection bias, we match respondents using only one of the three main drugs in pairs based on propensity scores, resulting in three matched samples, each of which containing two drug groups. Probit regression model is used in the propensity score matching (PSM) process, with three binary variables indicating the type of drug use as the dependent variables: Y_h-m_, 0 = Heroin, 1 = Methamphetamine; Y_h-k_, 0 = Heroin, 1 = Ketamine; Y_m-k_, 0 = Methamphetamine, 1 = Ketamine. The main reason for applying PSM analysis is to control for the systematic differences of certain characteristics between treatment groups and hence avoid treatment selection bias. In causal analyses, we usually presuppose that participants are randomly imposed with different treatments. However, in most non-experimental studies, the treatments are actually imposed non-randomly, and there are certain factors that affect both the outcomes and the process of treatment assignment. The non-randomization of treatment can lead to a biased estimation of the treatment effect, and cannot be dealt with simply by adding such factors as control variables.

In our study, the distribution of treatments, i.e., the type of drug a respondent is exposed to, is affected by the age of first drug use, time of first drug use and urban–rural status. However, these three factors also have an impact on the mental well-being of our respondents. First, due to differences in the social attributes of the three types of drugs in China, drug choice is closely related to the age of potential users. For instance, young people are more likely to be exposed to Ketamine than to be exposed to the other two types of drug. Since young persons in China are more vulnerable to mental health shocks (30), ignoring the systematically lower age of Ketamine user group might lead to overestimation of the negative impact of Ketamine on mental well-being. Second, the massive spread of these three types of drugs in China occurred at different times, with most Heroin users first exposed to drugs before 2005 and most Methamphetamine and Ketamine users first exposed in 2011–2015. Due to the accelerating pace of life and work in China in recent decades, overall mental health has gradually deteriorated (30). Therefore, Heroin users were exposed to drugs earlier and tend to have better baseline mental health than the other two groups of drug users, so neglecting such differences can lead to underestimation of the mental harm of Heroin. Third, affected by the varied spreading process of different drugs in China, a higher proportion of Ketamine users reside in urban areas than do the other two drug groups. The overall mental health of Chinese cities is higher than that of rural areas, mainly due to the advantage of economic level (30). Ignoring the systemic difference of urban–rural status across drug user groups may result in underestimation of the mental harm of Ketamine.

In addition, we included gender, educational attainment, occupation before using drug, and household registration city as independent variables in the PSM analysis. These independent variables are relatively fixed and unlikely to change much before and after drug use, so they can also be viewed as characteristics of respondents before treatment. It’s notable that we only included respondents who only used one of the three major drugs in the PSM analysis. The reason for excluding users of mixed drugs is to reduce complexity. In fact, both literature and field research indicate that usage of mixed drugs in China is usually transitional and short-term (31). After the PSM process, multilevel linear regression models with random intercepts are applied to predict BSI score; except for replacing the four-category drug type variable to the three dichotomous variables measuring drug type, the other independent variables are consistent with the regression Model 2 mentioned in 2.3.1.

#### 2.3.3 Moderated mediation model

**Figure 1:**
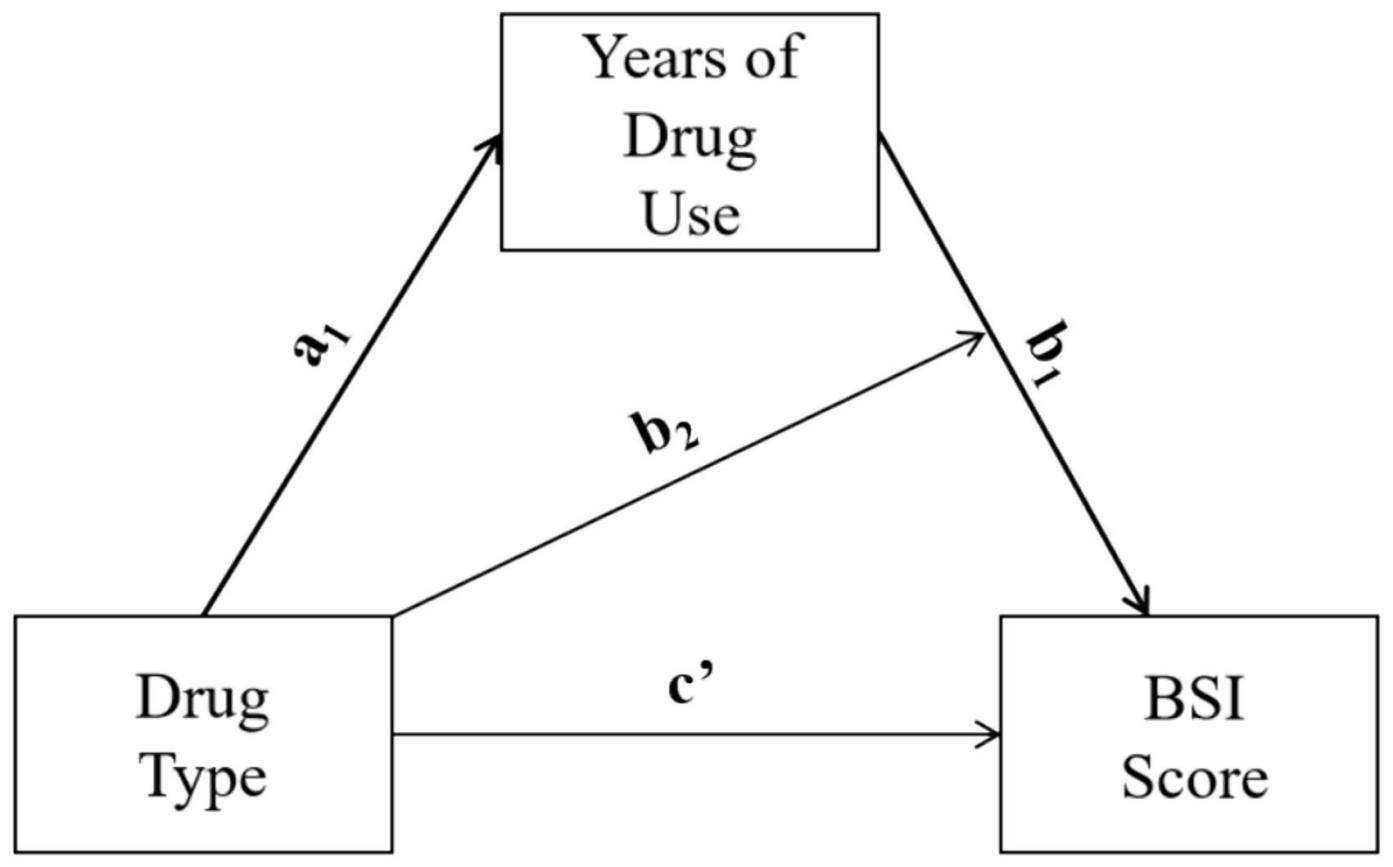
Conceptual model of the moderating-mediate effect of drug type.

We analyze the moderating-mediate effect of drug types on mental health through applying mediation analysis on the three propensity score matched samples. Based on literature and prior analysis, this study hypothesizes that there is a moderating mediating effect between drug type, duration of drug use, and mental health (see Figure 1). Drug type has a significant impact on the duration of drug use, and different drugs have different effects on mental health within the unit drug-use time. Therefore, referring to moderated-mediation model type 1 (32), we evaluate the strength of the moderating and mediating effects using the following equations:


(1) $$M=a_{0}+a_{1}X+a_{i}Z_{i}+\varepsilon_{1}$$

(2) $$Y=b_{0}+c^{’}X+\left(b_{1}+b_{2}X\right)M+b_{i}Z^{’}i+\varepsilon_{2}$$

In Equation 1, dependent variable is the drug-use duration *M*, *X* indicates one of the three drug-type dichotomous variables, and Z_i_ indicates the other independent variables.^2^ In Equation 2, *Y* indicates the BSI score, while the independent variables include a drug-type indicator *X*, drug-use duration *M* (i.e., the mediating variable), the interaction term between drug type and drug-use duration (i.e., observed moderating effect), and other variables Z’_i_.^3^ Conditional indirect effect of *X* on *Y* can be demonstrated with the following equation:


$$f\left(\hat{\theta }\mathrm{|X}\right)={\hat{a}}_{1}\left({\hat{b}}_{1}+{\hat{b}}_{2}X\right)$$

where ${\hat{a}}_{1}$ , ${\hat{b}}_{1}$ and ${\hat{b}}_{2}$ are the predicted values of a_1_, b_1_ and b_2_.

## 3 Findings

Among the 6,906 respondents, there are 1,561 who only used Heroin, 2,937 who only used Methamphetamine, and 707 who only used Ketamine (Table 1). People who only used one drug account for 75.4% of the total sample. The average BSI score for people who only used Heroin is 22.24, for people who only used Methamphetamine is 21.82, and for people who only used Ketamine is 22.2. According to the results of two-sample *t*-test, the difference between Heroin and Ketamine is significant at 0.05 level, the difference between Heroin and Methamphetamine is marginally significant at 0.1 level, and there is no significant difference between Methamphetamine and Ketamine. Respondents who only used Heroin have an average age of 43.7, which is significantly higher than the other two groups. Respondents who only used Ketamine have the highest average years of education (9.78 years). The duration of drug use for respondents who only used Heroin is 7.18 years, and the duration of since last drug use for them is 8.12 years, both much longer than the other two groups.

**Table 1:** Descriptive statistics.

	Heroin only (*n* = 1,561)		Methamphetamine only (*n* = 2,937)		Ketamine only (*n* = 707)		Whole sample (*n* = 6,906)	
	Mean	SD	Mean	SD	Mean	SD	Mean	SD
BSI-18 score	22.24	9.37	21.82	8.29	21.39	8.23	22.20	9.03
Age	43.70	8.15	35.37	8.22	32.91	7.41	37.46	9.00
Years of education	8.23	2.29	8.64	2.57	9.78	2.45	8.73	2.58
Income (unit 1,000Yuan)	3,069	2,847	3,779	3,132	4,504	3,556	3,673	3,169
Child number	1.47	1.27	1.40	1.48	0.92	1.08	1.31	1.36
Family member number	3.62	1.86	4.00	2.07	3.61	1.79	3.78	1.98
Drug-use duration (year)	7.18	7.28	1.88	2.95	1.71	2.70	3.75	5.47
Years since last drug use	8.12	5.84	4.86	3.06	5.82	3.85	5.88	4.44

	Heroin only	Methamphetamine only	Ketamine only	Whole sample
Male	95.9%	90.5%	84.3%	91.7%
Rural	42.2%	58.0%	28.1%	48.1%
No job before drug use	32.4%	30.3%	30.5%	31.4%
Marriage				
Single	23.2%	35.7%	41.5%	33.6%
Married	64.6%	56.2%	49.7%	56.6%
Divorced	12.2%	8.0%	8.8%	9.9%
Age of first drug use				
20 and less	17.5%	13.4%	26.2%	17.6%
21–30	44.2%	49.2%	52.3%	48.3%
31–40	28.6%	27.4%	16.1%	25.5%
41 and more	9.7%	10.0%	5.4%	8.6%
Year of first drug use				
2005 and before	49.1%	5.2%	6.6%	19.7%
2006–2010	24.3%	11.5%	18.5%	18.0%
2011–2015	17.5%	50.8%	50.4%	38.4%
2016–2020	9.1%	32.5%	24.5%	23.8%

Significance as follows: *p < 0.05, **p < 0.01, ***p < 0.001.

The proportion of male among people who use Heroin is as high as 95.9%, followed by those who use Methamphetamine (Table 1). The proportion of rural residents is 42.2% among people who use Heroin, 58% among people who use Methamphetamine, and 28.1% among people who use Ketamine. The respondents who only used Ketamine have the highest proportion of first-time drug use at age 20 and below (26.2%), which is much higher than the other two groups. The proportion of people who only used Heroin or Methamphetamine to begin using drug at age 41 and above is nearly twice that of people who only used Ketamine. Nearly half of the respondents who used Heroin started using drug before 2005, while nearly half of the respondents who used Methamphetamine or Ketamine started using drugs between 2011 and 2015.

As is shown in Table 2 Model 1, BSI score rises for 0.064 as the age of a respondent increases by 1 year, indicating a negative effect of aging on mental health, while the years of education, personal monthly income, and the duration since the last drug use have protective effects. Compared to the respondents who only use Heroin, the respondents who only use Methamphetamine tend to have lower BSI scores (*B* = −0.705, SE = 0.340, *p* < 0.05). The difference of BSI score between respondents who use Heroin and Ketamine is not statistically significant (*B* = −0.082, SE = 0.450, *p* = 0.856). Results of Model 2 show that, after controlling for the duration of drug use, the difference of the effect between Heroin use and Methamphetamine use on BSI score is no longer statistically significant (*B* = 0.264, SE = 0.376, *p* = 0.483), while the BSI scores of respondents who only use Ketamine become significantly higher than that of people who only use Heroin (*B* = 0.966, SE = 0.493, *p* < 0.05). The effects of age, years of education, and years since last drug use are no longer significant, while duration of drug use has a significant negative effect on mental health (*B* = 0.279, SE = 0.036, *p* < 0.001). Model 3 adds the interaction terms of drug types and duration of drug use on the basis of Model 2. The effects of duration of drug use (*B* = 0.234, SE = 0.048, *p* < 0.001) on mental health remain to be statistically significant. The interaction term of only Ketamine and duration of drug use has a significant effect, indicating that respondents who only use Ketamine have an additional BSI score increase of 0.47 for every year of drug use comparing to those who only use Heroin.

**Table 2:** Results of 3-level linear regression models predicting BSI score.

	Model 1		Model 2		Model 3	
	*B*	SE	*B*	SE	*B*	SE
Age	0.071***	0.017	0.035	0.019	0.034	0.019
Male	−0.263	0.426	−0.475	0.464	−0.494	0.463
Marriage (single as reference)						
Married	−0.216	0.339	−0.157	0.370	−0.143	0.37
Divorced	0.176	0.449	−0.082	0.481	−0.081	0.481
Child number	−0.272*	0.132	−0.169	0.145	−0.173	0.145
Family member number	−0.080	0.067	−0.143*	0.073	−0.148*	0.073
Years of education	−0.131*	0.052	−0.099	0.057	−0.101	0.057
Income (Unit: 1,000 Yuan)	−2.902***	0.385	−2.893***	0.421	−2.873***	0.421
Years since last use	−0.121***	0.028	−0.076*	0.030	−0.077*	0.030
Drug type (“Only Heroin” as reference)						
Only Methamphetamine	−0.705*	0.340	0.264	0.376	0.105	0.465
Only Ketamine	−0.082	0.450	0.966*	0.493	0.028	0.616
Other	0.388	0.360	0.880*	0.384	0.421	0.495
Drug-use duration (year)			0.279***	0.036	0.234***	0.048
Drug type*Drug-use duration (“Only Heroin” as reference)						
Only Methamphetamine					−0.007	0.092
Only Ketamine					0.470*	0.191
Other					0.105	0.073
Constant	23.408***	1.101	23.096***	1.207	23.438***	1.223
Log likelihood	−16,170		−13,780		−13,776	
*N*	4,660		4,660		4,660	

Significance of two sided *t*-test as follows: *p < 0.05, **p < 0.01, ***p < 0.001.

After the Propensity Score Matching process, the relative multivariate imbalance measure shrank from 0.322 to 0.002 for the Heroin-Methamphetamine sample, from 0.083 to 0.001 for the Heroin-Ketamine sample, and from 0.328 to 0.016 for the Methamphetamine-Ketamine sample. Through propensity score matching, the age of first drug use, year of first drug use and urban–rural status between drug user groups have in general been reduced significantly (Table 3). The only two exceptions are the difference of the age of first drug use for Heroin and Methamphetamine sample and the proportion of users with first drug use between 2011 and 2015 for Methamphetamine and Ketamine sample. Yet the differences of these two variables in specific samples were extremely small even before sample matching (with biases of 0.7 and 0.6%), so even though these differences increased after PSM, it would not significantly increase sample unbalance.

**Table 3:** Balance diagnose for propensity score matching.

	Heroin and Methamphetamine			Methamphetamine and Ketamine			Heroin and Ketamine		
	*H*	*M*	Reduced bias	*M*	*K*	Reduced bias	*H*	*K*	Reduced bias
Age of first drug use									
Unmatched	28.6	28.8	−290.4%	28.8	25.7	95.4%	28.6	25.7	60.1%
Matched	29.6	28.8		25.9	25.7		24.6	25.7	
Year of first drug use									
2006–2010									
Unmatched	23.8%	11.4%	94.2%	11.5%	19.1%	97.9%	23.8%	19.1%	29.9%
Matched	10.7%	11.4%		19.3%	19.1%		15.9%	19.1%	
2011–2015									
Unmatched	17.6%	50.9%	92.8%	50.9%	51.2%	−655.0%	17.7%	51.2%	87.3%
Matched	48.5%	50.9%		49.1%	51.2%		47.0%	51.2%	
2016–2020									
Unmatched	9.2%	32.6%	86.0%	32.6%	24.1%	90.4%	9.2%	24.1%	85.7%
Matched	35.9%	32.6%		24.9%	24.1%		26.2%	24.1%	
Rural									
Unmatched	42.5%	57.8%	91.4%	58.0%	29.5%	96.0%	42.6%	29.5%	96.3%
Matched	59.2%	57.8%		30.6%	29.5%		30.0%	29.5%	

The results of the 3-level linear regression on the samples matched based on propensity score are shown in Table 4. In the Heroin-Methamphetamine sample, the interaction term of drug type and duration of drug use has no significant effect on BSI score, indicating that influence of Heroin use and Methamphetamine use on mental health per unit time is similar. Effects of the interaction terms on mental health in the other two samples are significant at 0.001 level. For each year of drug use, Ketamine leads to 0.6 additional increase of BSI score comparing to Heroin, and 0.55 additional increase of BSI score comparing to Methamphetamine. Such results indicate that the per unit time negative impact of Ketamine on mental health is greater than the other two drugs. Results of regression models predicting the scores of three sub-scales of BSI-18 as dependent variables are consistent with the analysis of the total BSI score (see Table 5), indicating that within the same duration of drug use, Ketamine causes higher risk of somatization (compare to Heroin: *B* = 0.225, *p* < 0.001; compare to Methamphetamine: *B* = 0.230, *p* < 0.001), depression (compare to Heroin: *B* = 0.163, *p* < 0.001; compare to Methamphetamine: *B* = 0.145, *p* < 0.01), and anxiety (compare to Heroin: *B* = 0.190, *p* < 0.001; compare to Methamphetamine: *B* = 0.163, *p* < 0.01).

**Table 4:** Results of 3-level linear regression predicting BSI score for propensity score matched samples.

	Heroin and Methamphetamine		Heroin and Ketamine		Methamphetamine and Ketamine	
	*B*	SE	*B*	SE	*B*	SE
Age	0.000	0.021	0.057	0.030	−0.014	0.023
Male	0.184	0.546	0.093	0.747	−0.144	0.488
Marriage(single as reference)						
Married	−0.469	0.422	−0.775	0.568	−0.528	0.431
Divorced	0.027	0.559	−1.152	0.729	−0.186	0.614
Child number	−0.030	0.158	−0.386	0.231	0.094	0.168
Family member number	−0.088	0.080	−0.005	0.124	−0.094	0.083
Years of education	0.055	0.064	0.079	0.090	0.011	0.068
Income (Unit: 1,000Yuan)	−0.225***	0.047	−0.291***	0.067	−0.163***	0.048
Years since last use	−0.015	0.035	−0.094	0.041	−0.007	0.047
Drug-use duration(year)	0.230***	0.036	0.206***	0.038	0.251***	0.060
Methamphetamine comparing to Heroin	0.055	0.448	–	–	–	–
Ketamine comparing to Heroin	-	–	−0.308	0.618	–	–
Ketamine comparing to Methamphetamine	–	–	–	–	−0.277	0.473
Drug type*Drug-use duration	0.015	0.069	0.603***	0.128	0.552***	0.134
Constant	21.502***	1.303	20.518***	1.802	22.366***	1.280
Log likelihood	−10,085.686		−5,172.4941		−8,171.2821	
*N*	2,949		1,516		2,415	

Significance of two sided *t*-test as follows: *p < 0.05, **p < 0.01, ***p < 0.001.

**Table 5:** Results of 3-level linear regression predicting scores of sub-scales of BSI on matched samples.

	Heroin and Methamphetamine		Heroin and Ketamine		Methamphetamine and Ketamine	
	*B*	SE	*B*	SE	*B*	SE
Somatization						
Drug-use duration(year)	0.078***	0.012	0.065***	0.013	0.067***	0.019
Drug B	0.055	0.153	−0.092	0.219	−0.227	0.153
Drug B*Drug-use duration	−0.016	0.024	0.225***	0.045	0.230***	0.044
Depression						
Drug-use duration(year)	0.092***	0.013	0.085***	0.014	0.102***	0.022
Drug B	0.079	0.166	−0.045	0.232	−0.037	0.177
Drug B*Drug-use duration	0.009	0.026	0.163***	0.048	0.145**	0.050
Anxiety						
Drug-use duration(year)	0.067***	0.013	0.056***	0.013	0.083***	0.022
Drug B	−0.067	0.159	−0.061	0.219	0.024	0.171
Drug B*Drug-use duration	0.017	0.025	0.190***	0.045	0.163**	0.049

Significance of two sided *t*-test as follows: *p < 0.05, **p < 0.01, ***p < 0.001.

Table 6 shows the results of mediation analysis using the moderated mediation effect models. Conditional indirect effects are examined through generating 5,000 Bootstrap samples, which is a test recommended by prior studies on moderated mediation effect (32). The models for the three sub-samples use different dichotomous variables as *X* to indicate drug types (*X* for H-M sub-sample: 0 = Heroin & 1 = Methamphetamine; X for H-K sub-sample: 0 = Heroin & 1 = Ketamine; X3: 0 = Methamphetamine & 1 = Ketamine). M represents the duration of drug use, MX represents the interaction term between *X* and *M*, and *Y* represents BSI score.

**Table 6:** Results of mediation analysis for propensity score matched sub-samples (5,000 bootstraps).

Sub-samples	X- > M	M- > Y	MX- > Y	Direct effect	Conditional indirect effect
H-M	−0.699***	0.237***	0.047	−0.065	−0.164**
	(SE = 0.177)	(SE = 0.038)	(SE = 0.071)	(SE = 0.442)	(SE = 0.056)
H-K	−1.657***	0.259***	0.302***	0.028	−0.369***
	(SE = 0.325)	(SE = 0.042)	(SE = 0.066)	(SE = 0.625)	(SE = 0.092)
M-K	−0.541***	0.264***	0.595***	0.140	−0.206**
	(SE = 0.124)	(SE = 0.062)	(SE = 0.136)	(SE = 0.471)	(SE = 0.066)

Significance of two sided t-test as follows: *p < 0.05, **p < 0.01, ***p < 0.001.

Control variables are also included in mediation analysis but not presented in this table.

In all three models, the direct effects of drug type on the duration of drug use and the direct effects of duration of drug use on BSI are statistically significant, while the direct effect of drug type on BSI is not significant at 0.05 level (Table 6). Model 1 shows that Methamphetamine leads to 0.699 years shorter of drug-use duration than Heroin, while its moderating effect is not statistically significant. For the H-M sub-sample, the conditional indirect effect of drug type through the duration of drug use on BSI score is −0.164 and significant at 0.01 level. Model 2 shows that Ketamine leads to 1.657 years shorter of drug-use duration than Heroin, but also leads to 0.302 extra increase of BSI score for each year of drug use comparing to Heroin. For the H-K sub-sample, the conditional indirect effect is −0.369 and significant at 0.001 level. Model 3 shows that Ketamine leads to 0.541 years shorter of drug-use duration than Methamphetamine, but also leads to 0.595 extra increase of BSI score for each year of drug use comparing to Heroin. For the M-K sub-sample, the conditional indirect effect is −0.206 and significant at 0.01 level.

**Figure 2:**
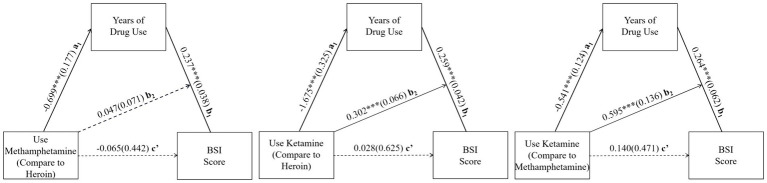
Moderated mediation models for BSI score (solid line: *p* < =0.05; dotted line: *p* > 0.05).

The results of mediation analysis are presented in Figure 2. To sum up, for H-M sub-sample, drug type has an indirect effect on BSI score with duration of drug use as mediating factor; for H-K and M-K sub-samples, drug type has a moderating effect on duration of drug use while duration of drug use serves as a mediating factor between drug type and BSI score. These results indicate that Ketamine can cause higher damage to mental damage than do Heroin and Methamphetamine, but is correlated with shorter duration of drug use. This is probably because Ketamine consumption is highly socialized and easily exposed (often consumed in Karaoke or bars), making it a priority target for the anti-drug authorities. Mediation analysis in this study also shows that Heroin and Methamphetamine do similar harms to mental well-being per unit time of drug use, even though they seem to affect users in rather different ways.

## 4 Discussion and conclusion

This study reveals a mediation-moderating effect of types of drugs on the correlation between duration of drug use and mental health (Figure 2). It systematically compared the differences in the impact of three main drugs on mental health in a society like China where drug control is strict. When no variables were controlled, Heroin use is associated with the severest mental health issues. The average duration of Methamphetamine use is shorter than that of Heroin use, but people with the same duration of drug use of these two types of drugs tend to have the similar risks of mental health issues. The average duration of Ketamine use is shortest among the three major types of drug, but after the same time of drug use, using Ketamine is associated with worse mental health than that using Heroin or Methamphetamine. Overall, there is a clear negative association between the use of these three drugs and mental well-being, calling for more attentions paid to the mental health of people through drug rehabilitation process regardless.

In addition, this study found that income has a protective effect on the mental health of people who use drugs in the original data and in all three PSM sub-samples. This once again demonstrates that the impact of drug on mental health of people who use drugs is not solely biological, but also through influencing their social relationship (33). People who use drugs might experience guilt toward their families due to a partial or complete loss of earning ability, leading to exclusion from their families, communities and the society. These are important risk factors for psychological issues among them. In particular, Heroin use often leads to loss of work capacity, making unemployment a particularly serious issue. Therefore, it can be inferred that the measures taken by many countries to assist people who use drug in finding employment will also have a positive effect on safeguarding their mental health.

Through studying a country with strict drug control like China, we can also to some extent infer the possible changes in the impact of drugs in other social contexts. Heroin is highly addictive, and the physiological withdrawal symptoms are very painful, making it difficult to quit through personal will power alone. Short-term use of Ketamine can cause significant damage to mental health. In a society with looser drug control, the duration of Ketamine use is likely to far exceed that of a society with strict drug control, resulting in serious psychological harm. Therefore, in societies that are relatively more tolerant of drug use, more attention should be paid to providing accessible psychological health service for people who use drugs. The psychological impact of Methamphetamine should not be underestimated, as literature suggests that long-term use of Methamphetamine may lead to personality changes, causing people to be unaware of the harm that drugs have on themselves (34). Self-assessment mental health scales tend to have lower validity for people who use Methamphetamine, as Methamphetamine use has been observed to lead to self-deception, overconfidence, and blame-shifting (35, 36). Future research should consider using a variety of different measurement methods, especially peer assessment scales, to more accurately assess the psychological impact of Methamphetamine use.

The main limitation of this study is the lack of baseline mental health scores before drug use, since prior studies clearly show that the prospective risk of drug use initiation is associated with poor mental health (37–39). To reduce the systematic differences before drug use across drug types, this study used PSM to achieve a more balanced distribution in terms of gender, SES, and urban–rural areas. However, the initial mental health status of individuals using different types of drugs may have systematic differences, which could still interfere with data analysis. Moreover, the majority of respondents in this study are distant from the periods of active drug use, so the mental damage caused by drug has mitigated over time and the data only reflect their current mental health status. In addition, this study excluded respondents who used mixed drugs in both PSM and mediation analyses, which may lead to an incomplete analysis of the effects of drugs. The reason for excluding people who used mixed drugs is that including multiple categories of people who use mixed drugs would make the model too complex and not conducive to analyzing the effects of different types of drugs. Future research can contribute to the field through further analyzing the impact and mechanisms of using mixed drugs.

## Data Availability

The raw data supporting the conclusions of this article will be made available by the authors, without undue reservation.

## Footnotes

^1^ICC measures the relatedness of cases within clusters, and mathematically it equals to the ratio of within-cluster variances to total observed variances (28). While some scholars argue that multi-level regression analysis has no advantage over one-level regression analysis when ICC is small, (29) presents several cases where ICCs are small but multilevel analysis is necessary, and notes that multi-level analyses can be applied when three criteria are met: first, conceptual structure is clear; second, there are enough data for each cluster; third, lower level units can be classified into specific higher level units.

^2^Other independent variables Z_i_ in model I include: age of first drug use, year of first drug use, years of education, urban–rural binary variable, and city indicator.

^3^Other independent variables Z’_i_ in model II include: age of first drug use, years of education, urban–rural binary variable, city variable, personal monthly income, marital status, number of children, and number of family members.
